# A survey of genome-wide association studies, polygenic scores and UK Biobank highlights resources for autoimmune disease genetics

**DOI:** 10.3389/fimmu.2022.972107

**Published:** 2022-08-05

**Authors:** Rochi Saurabh, Césaire J. K. Fouodo, Inke R. König, Hauke Busch, Inken Wohlers

**Affiliations:** ^1^ Medical Systems Biology, Lübeck Institute for Experimental Dermatology (LIED) and Institute for Cardiogenetics, University of Lübeck, Lübeck, Germany; ^2^ Institute of Medical Biometry and Statistics (IMBS), University of Lübeck, Lübeck, Germany

**Keywords:** autoimmune disease, genetics, genome-wide association study, GWAS, polygenic scores, genetic risk, UK Biobank, experimental factor ontology

## Abstract

Autoimmune diseases share a general mechanism of auto-antigens harming tissues. Still. they are phenotypically diverse, with genetic as well as environmental factors contributing to their etiology at varying degrees. Associated genomic loci and variants have been identified in numerous genome-wide association studies (GWAS), whose results are increasingly used for polygenic scores (PGS) that are used to predict disease risk. At the same time, a technological shift from genotyping arrays to next generation sequencing (NGS) is ongoing. NGS allows the identification of virtually all - including rare - genetic variants, which in combination with methodological developments promises to improve the prediction of disease risk and elucidate molecular mechanisms underlying disease. Here we review current, publicly available autoimmune disease GWAS and PGS data based on information from the GWAS and PGS catalog, respectively. We summarize autoimmune diseases investigated, respective studies conducted and their results. Further, we review genetic data and autoimmune disease patients in the UK Biobank (UKB), the largest resource for genetic and phenotypic data available for academic research. We find that only comparably prevalent autoimmune diseases are covered by the UKB and at the same time assessed by both GWAS and PGS catalogs. These are systemic (systemic lupus erythematosus) as well as organ-specific, affecting the gastrointestinal tract (inflammatory bowel disease as well as specifically Crohn’s disease and ulcerative colitis), joints (juvenile ideopathic arthritis, psoriatic arthritis, rheumatoid arthritis, ankylosing spondylitis), glands (Sjögren syndrome), the nervous system (multiple sclerosis), and the skin (vitiligo).

## Introduction

Autoimmune diseases are a range of diseases in which the immune response to self-antigens results in damage or dysfunction of tissues. It can be systemic or can affect specific organs or body systems. Autoimmune diseases are characterized by a multifactorial etiology, in which genetic factors interplay with environmental factors. Estimates of heritability, that is, variability in occurrence of autoimmune disease explained by genetic factors, vary considerably and have been reported to be between 42 and 91% for pediatric age-of-onset and lower for adult onset cases (1). Variation in the human major histocompatibility complex (MHC) regions harboring the human leukocyte antigen (HLA) genes is most strongly linked to autoimmune disease (2). Beside HLA, other genetic loci are shared between autoimmune diseases, with first investigations finding 47/107 (44%) immune-mediated disease risk variants associated with multiple, but not all such diseases (3), and later work identifying 244 shared disease loci (4). Accordingly, efforts are ongoing to unravel shared disease mechanisms based on shared genetic profiles (5, 6). Such genetics-driven systems approaches to autoimmune disease can largely benefit from public resources of genome and phenotype data as well as derived information. Here we perform a survey of autoimmune disease-related content in three such resources: (i) The NHGRI-EBI GWAS catalog (7) reporting on genome-wide association studies (GWAS), (ii) the newly established PGS catalog (8) having information on polygenic scores (PGS) and (iii) the UK Biobank (UKB) holding genetic and phenotypic data of ~500,000 people from the UK (9) (for details on data processing, see https://github.com/iwohlers/2022_autoimmune_review). While GWAS aim to identify associations of a large number of genetic variants with phenotypes or traits (10), the main goal of PGS studies is to estimate the risk of developing a disease or of the presence of a specific trait depending on the genetic profiles (11).

## Autoimmune diseases and their relationships within biomedical ontologies

An ontology is a controlled vocabulary, formalizing domain knowledge into terms and their relationships. The Experimental Factor Ontology (EFO) is a biomedical ontology curated by the European Bioinformatics Institute (EBI) and used by the GWAS and PGS catalog for the purpose of disease classification (12). The part of EFO relating to autoimmune disease is shown in **Suppl. Table S1** (EFO version 3.42.0; https://bioportal.bioontology.org/ontologies/EFO). It contains “is a” relationships between “parent” and “child” terms, e.g., rheumatoid arthritis (child term) is an autoimmune disease (parent term). The ontology branch of child terms for autoimmune disease contains 120 terms organized in up to five levels (**Suppl. Table S1**). For the disease-related part of EFO, some terms have been taken from other ontologies (denoted by IDs not starting with “EFO”). Within the autoimmune disease sub-branch, 13 terms are taken from the Mondo disease ontology that is also curated by EBI [https://bioportal.bioontology.org/ontologies/MONDO; (13)]. Further, three terms are taken from Orphanet, an online database of rare diseases and orphan drugs (Copyright, INSERM 1997. available at http://www.orpha.net) and one term from the disease ontology [www.disease-ontology.org; (14)], cf. **Suppl. Table S2**.

## Genomics data, genetic variation and notable reference resources

Differences between genomic sequences are called genetic variations. They are classified into single nucleotide variants (SNVs), for which the base at a single position differs, indels, which are insertions or deletions of size up to 50 bases, and structural variants (SVs), which are genomic alterations of a size larger than 50 bases. SNVs commonly found in a population are also called single nucleotide polymorphisms (SNPs). Genotyping arrays assess predefined, common variants, i.e., SNPs. Genetic variations are specified with respect to a specific reference genome. For humans, this is GRCh38, the genome of the Genome Reference Consortium, with its latest version 38.

Human SNVs have been well characterized. The first milestone was the whole genome sequencing performed as part of the 1000 Genomes Project, which resulted in “a global reference of human genetic variation” based on the genomic data of 2,504 individuals from 26 populations (15). The 1000G-based genetic variation with respect to the reference genome was overall 84.7 million SNVs, 3.6 million indels and ~60,000 structural variants; each individual carried 4.1 million to 5.0 million sites that differed from the reference genome. This first comprehensive catalog of genetic variation was later extended by whole genome sequencing of the Human Genome Diversity Project (n=929) (16). Of the many, often national, genome sequencing initiatives, gnomAD [n=71,702 whole genome sequenced; (17)], Topmed [n=53,831 whole genome sequenced; (18)] and the UK Biobank [UKB, n=150,119 whole genomes sequenced; (19)] stand out in terms of sample size. Within the UKB cohort, 585.0 million SNVs, representing 7.0% of all possible human SNVs, 58.7 million indels and ~900,000 SVs have been identified (19). Many of the SNVs, 46%, are carried by only one sequenced individual (called “singletons”) and only 3.4% (~20 million) have a frequency of more than 0.1% (19).

## Autoimmune disease genome-wide association studies (GWAS)

The main goal of GWAS is to identify associations of genetic variants with a phenotype or trait without prior knowledge about their genomic location. Although GWAS could in principle use different kinds of genetic variants, to date almost always SNPs are utilized (11). GWAS then consist of testing for associations of SNPs with the phenotype or trait of interest. Since the first GWAS about twenty years ago (20), more than 5,700 analyses have been conducted, yielding more than 3,300 traits established to be statistically associated with genetic variants (10).

Testing for associations between a phenotype or a trait and genetic variants is based on a statistical model, and the type of the model used depends on whether the phenotype or trait is continuous (e.g. Body Mass Index (BMI)) or dichotomous (e.g. presence or absence of an autoimmune disease). In the case of a continuous phenotype or trait, a linear regression model is most commonly used, whereas logistic regression is mostly applied for dichotomous ones. Typically, the models are estimated for each single variant separately. The typical GWAS output comprises, for each variant, a report giving the ID of the variant according to dbSNP (21), the effect allele, the statistical effect and the corresponding p-value. Since GWAS test a large number of genetic variants at the same time, the statistical significance threshold has to be corrected to avoid false positive results. The widely used approach for this aim is the so-called Bonferroni correction (10, 22), consisting of dividing the overall statistical significance threshold by the total number of independent tests, in this case, the tested independent variants. As a consequence, a threshold of 5*10^-8^ is commonly used in practice, since the human genome contains approximately one million common, independent variants (10).

The GWAS catalog is a publicly available, manually curated resource, which contains published GWAS and association results and is developed by the NHGRI and EMBL-EBI (7). Catalog data is provided for the latest reference genome version (GRCh38.p13) and variant database version (dbSNPBuild 154). GWAS catalog source files of studies and associations have been used in our survey (files gwas-catalog-studies_ontology-annotated.tsv and gwas-catalog-associations_ontology-annotated.tsv from http://ftp.ebi.ac.uk/pub/databases/gwas/releases/2022/05/23/), and entries with “MAPPED_TRAIT_URI” an autoimmune EFO IDs (**Suppl. Table S2**) were extracted. Overall, the GWAS catalog studies contain 442 autoimmune disease GWAS (“STUDY ACCESSION”) published between 2006 and 2022 in 58 different journals with 221 unique PubMed IDs (**Suppl. Table S3**); these studies have been conducted on 377 different datasets (according to column “INITIAL SAMPLE SIZE” in **Supplementary Table S3**). A subset of studies (n=179 (47%)) reported no genome-wide significant variants.

The GWAS catalog contains 5,023 associations that cover 41 autoimmune diseases (according to EFO ID) based on 253 datasets (according to column “INITIAL SAMPLE SIZE”) relating to 200 unique PubMed IDs (**Suppl. Table S4**). These associations correspond to 3,212 unique SNPs (according to column “SNPS”) and 1,760 unique genes or gene combinations reported in the literature (column “REPORTED GENE(S)”; **Supplementary Table S4**).

## Polygenic scores (PGS) developed for autoimmune diseases

GWAS are typically used for traits with an underlying polygenic architecture, that is, many genetic variants just show small effect sizes on the phenotype or trait of interest. As a result, prediction performances of single associated variants are generally poor. Therefore, polygenic scores, also termed “risk scores” if applied to a disease, are used to overcome these limitations. The main idea of predictive models based on polygenic scores is to combine effects of single genetic variants to expect a stronger association with the response phenotype or trait. The standard approach used for quantifying genetic liability in the prediction of disease risks are weighted polygenic scores (11). Based on this, PGSs are generally obtained as weighted sum scores of risk alleles using effect sizes from GWAS. More recently, new statistical machine learning approaches have emerged as a powerful approach for the computation of PGS (23).

The PGS catalog is a database and website established with the aim of making published PGS easily available and allowing their systematic evaluation (8). We obtained PGS catalog source files from https://www.pgscatalog.org/downloads and extracted from the respective files *via* EFO IDs all information related to autoimmune diseases: polygenic scores (**Suppl. Table S5**), score development samples (**Suppl. Table S6**), performance evaluation metrics (**Suppl. Table S7**) and evaluation samples (**Suppl. Table S8**). The database contains 18 autoimmune diseases for which 47 polygenic scores are published in 14 papers between 2018 and 2022 (cf. **Suppl. Table S5**). These have been developed with 15 different computational methods, mostly with the tools snpnet [n=18 scores; (24)], using genome-wide significant GWAS variants (n=7 scores), LDPred [n=6; version 1 and 2; (25, 26)] or by applying pruning and threshold (n=4). Corresponding to the method or tool applied for PGS constructions, the number of variants considered in the scores ranges from 3 to 6,907,112. Many cohorts of mainly European and/or East Asian (largely Chinese) ancestry have been used for score development, mainly as source GWAS underlying the respective score, but also for parameter training (**Suppl. Table S6**). Further, a large number of PGS have been developed using the UK Biobank. Autoimmune PGS have been evaluated in 124 data sets (**Suppl. Table S7**) yielding 225 performance assessments in 16 publications (**Suppl. Table S8**). The most common performance measure is the Area Under the Receiver-Operating Characteristic Curve (AUROC), which shows the fraction of individuals incorrectly classified as having the disease (false positive rate) versus the fraction of individuals correctly classified as having the disease (true positive rate) at different PGS score thresholds. An AUROC value of 0.5 corresponds to a random and a value of 1.0 to a perfect classification. Typically, AUROC classification performance of autoimmune PGS are in the range from 0.56 to 0.99 (provided for n=124; **Suppl. Table S8**). Overall, according to the PGS catalog, 16 different publications either constructed and/or evaluated an autoimmune disease PGS (columns “PGS Publication (PGP) ID” of **Suppl. Table S5** and **Suppl. Table S8**).

## Genetic data and autoimmune diseases covered by the UK Biobank

The UK Biobank (UKB) is the largest resource for human genomic and phenotypic data available for global academic health research, containing data from approximately 500,000 individuals from the United Kingdom (9). Its first release in 2018 contained UK Biobank Axiom Array-based genotypes (m=825,927) imputed to m~96 million variants of these individuals (9). In 2021, whole exome sequencing data of 454,787 of its individuals was released (with m~2 million exonic SNVs) (27). In 2022 whole genome sequencing-based variants for n=150,119 individuals resulted in overall m~585 million SNVs, ~59 million indels, 2.5 million microsatellites and 900k structural variants (19), representing a nearly complete variant profile for these individuals.

UK Biobank provides medical diagnosis according to the International Statistical Classification of Diseases and Related Health Problems (ICD) of the World Health Organization (WHO), whose current version is ICD-10. Besides ICD-10 codes gathered from medical records, UK Biobank provides diagnoses that are self-reported by participants, referred to by dedicated UKB-internal IDs (starting with “20002_”). To extract autoimmune disease information from UK Biobank, we used the mapping file internal to the ontology mapping tool Zooma of the EMBL-EBI ontology lookup service (28) which was generated as part of a large-scale, comprehensive mapping of UK Biobank ICD-10 codes and self-reported diseases to EFO terms (https://github.com/EBISPOT/EFO-UKB-mappings). We find that 20 of 120 autoimmune EFO IDs (cf. **Suppl. Table S2**) correspond to patients and patient genotypes within UK Biobank (**Suppl. Table S9**), of which 9 have both self-reported and ICD-10 diagnosis, 6 are only self-reported and 5 only have ICD-10-based information. The number of respective patients ranges from 13 (reactive arthritis with ICD-10 code M03) to 12,556 (rheumatoid arthritis with ICD-10 code M06) (**Suppl. Table S9**).

## Overlap between autoimmune diseases assessed by GWAS, PGS and UKB

**Figure 1:**
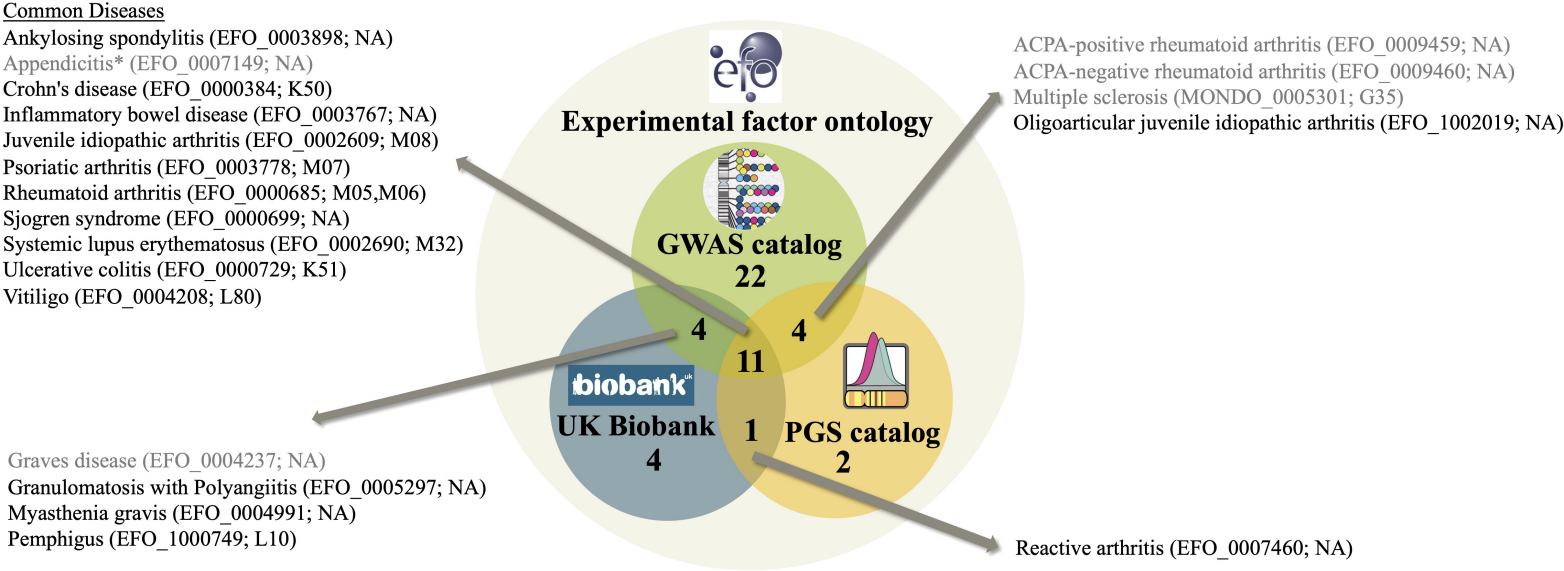
A Venn diagram representing the number of autoimmune diseases in the experimental factor ontology (EFO) overlapping with GWAS catalog and PGS catalog covered EFO IDs as well as UK Biobank data field matched according to **Suppl. Table S9**. EFO IDs of diseases in more than two resources are listed together with their name according to EFO and their UKB ICD-10 code according to **Suppl. Table S9**. Diseases shaded gray are affected by issues with disease definition and classification compromising the mapping. These issues are: (i) Appendicitis is classified as an autoimmune disease in EFO because it is a child term of inflammatory bowel disease, however, it is not considered an autoimmune disease. (ii) Grave’s disease EFO child terms are in PGS, not the EFO ID of Grave’s disease itself though. (iii) ACPA-positive and ACPA-negative rheumatoid arthritis is not mappable to UKB. UKB, however, contains seropositive and other rheumatoid arthritis, a distinction not covered by EFO. (iv) UKB has information on multiple sclerosis, yet since the recent EFO version was updated to using the MONDO ID for multiple sclerosis, the mapping to UKB data fields failed. “NA” denotes that mapping to UKB is not available for the respective EFO ID.

We investigated for which autoimmune diseases the GWAS catalog, the PGS catalog and UK Biobank contain information by comparing the respective autoimmune EFO IDs covered by each resource. Overall, there are 120 autoimmune disease EFO IDs (**Suppl. Table S2**) representing different levels of diagnosis (**Suppl. Table S1**). Of those, the GWAS catalog covers 41 EFO IDs (**Suppl. Table S4**) and the PGS catalog 18 EFO IDs (**Suppl. Table S5**). As the UK Biobank does not use EFO IDs, we used a published mapping of EFO IDs to UKB data fields instead, as described in the last section and provided in **Suppl. Table S9**. This assigned 20 EFO IDs to traits in UKB (**Suppl. Table S9**). The overlap of autoimmune diseases covered by the three resources is shown in **Figure 1**. Out of 120 EFO IDs, only 48 autoimmune diseases are present in any of the three databases, most in the GWAS catalog. The GWAS catalog is sharing 15 autoimmune disease EFO IDs with the PGS catalog and 15 can be mapped to UK Biobank. Further, 12 disease EFO IDs are shared between PGS catalog and UK Biobank. There are 11 autoimmune disease EFO IDs common to all three databases. They relate to: ankylosing spondylitis, appendicitis, Crohn’s disease, inflammatory bowel disease, juvenile idiopathic arthritis, psoriatic arthritis, rheumatoid arthritis, Sjögren syndrome, systemic lupus erythematosus, ulcerative colitis and vitiligo. Several of the autoimmune diseases related to EFO IDs that are not shared by all three resources are cases that highlight limitations with respect to the definition of terms and relationships within the EFO and in the mapping of EFO terms to external codes and identifiers, which may not be one-on-one and needs disease-specific knowledge (for details see caption of **Figure 1**).

We have investigated more closely the 10 autoimmune diseases with most GWAS studies according to GWAS catalog (**Table 1**). They are systemic lupus erythematosus (29), rheumatoid arthritis (30), multiple sclerosis (31), inflammatory bowel disease (32) with its two subtypes Crohn’s disease and ulcerative colitis, vitiligo (33), Sjögren syndrome (34), Grave’s disease (35), and Behcet’s syndrome (36). The GWAS catalog association data of the top 10 autoimmune diseases (underlying **Table 1**) are provided in **Supplementary Tables S10**-**S19**. The most GWAS-studied autoimmune disease is systemic lupus erythematosus, for which 37 different GWAS have been performed, the largest one using 13,377 cases and 194,993 controls. These studies have reported 788 unique SNPs and 439 unique genes or gene combinations. In the PGS catalog, six studies are noted on systemic lupus erythematosus, which report six different risk scores. These PGS have been evaluated in 32 settings. The largest number of cases has been analyzed for inflammatory bowel disease (n=25,042), the lowest for Sjögren syndrome (n=1,599). Overall, the number of independent genomic loci associated with disease increases with the number of studies and cases (**Table 1**).

**Table 1:** The ten autoimmune diseases (defined by EFO term) which have the highest number of GWAS studies registered at the GWAS catalog. Displayed is the summary of information obtained from GWAS catalog, PGS catalog and UK Biobank. With respect to GWAS catalog, this is the number of unique studies (according to column “STUDY ACCESSION” of **Suppl. Table S3**), the highest number of cases with corresponding number of controls, the number of unique variants reported (according to column “SNP_ID_CURRENT” of **Suppl. Table S4**), the number of independent, associated genomic loci reported in the literature, the number of unique genes or gene combinations reported in the respective publications (according to column “REPORTED GENE(S)” of **Suppl. Table S4**). With respect to PGS catalog, reported are the number of unique studies (according to column “PGS Publication (PGP) ID” of **Suppl. Table S5** and **Suppl. Table S8**), unique scores developed (according to column “Polygenic Score (PGS) ID” of **Suppl. Table S5**), the range of variants utilized in the scores for the respective disease and the number of performance evaluations in independent samples (according to column “PGS Performance Metric (PPM) ID” of **Suppl. Table S8**). Finally, for the UK Biobank, the UKB data field, ICD-10 code (if available in UKB) and patient number according to **Suppl. Table S9** is provided.

Trait	EFO IDs	GWAS catalog						PGS catalog				UK Biobank		
		# Studies	# Cases	# Controls	# SNPs	# Loci	# Gene	# Studies	# PGS	# Variants	# PGS Eval.	Data Field	ICD10 Code	# Indiv.
Systemic lupus erythematosus	EFO_0002690	37	13,377	194,993	788	132^1^	439	6	6	41- 293,684	32	131894	M32	1,053
Rheumatoid arthritis	EFO_0000685	37	22,628	288,664	421	>150^2^	249	3	6	3- 95,083	33	131850	*M06	12,556
Multiple sclerosis	MONDO_0005301	27	14,802	26,703	603	233^3^	479	3	5	36- 129,077	25	131042	G35	2,518
Crohn’s disease	EFO_0000384	27	12,924	21,442	411	>200^4^	265	1	2	220-257	9	131626	K50	3,355
Ulcerative colitis	EFO_0000729	25	12,366	33,609	295	>200^4^	184	2	4	179-566,637	26	131628	K51	6,451
Inflammatory bowel disease	EFO_0003767	12	25,042	34,915	387	>200^4^	238	3	2	195-690,7112	7	_**	_**	_**
Vitiligo	EFO_0004208	10	2,853	37,405	91	49^5^	80	3	3	42-77	10	131802	L80	1,201
Sjogren syndrome	EFO_0000699	10	1,599	658,316	48	25^6^	42	1	1	7	5	20002_1382	_	572***
Grave’s disease	EFO_0004237	8	4,487	629,598	74	12^7^	27	_	_	_	_	20002_1522	_	183***
Behcet’s syndrome	EFO_0003780	8	3,197	5,785	40	21^8^	35	_	_	_	_	41202	_	18****

^1^ (29); ^2^ (30); ^3^ (31); ^4^ (32); ^5^ (33); ^6^ (34); ^7^(35); ^8^ (36); *Excludes seropositive rheumatoid arthritis (M05) with 1,401 patients ** K50+K51 *** Self-reported **** Based on medical history of hospital patients.

## Discussion

We systematically reviewed the autoimmune disease-related content of the GWAS catalog of variant associations, the PGS catalog of polygenic scores and the UK Biobank of genomic and phenotypic data. These curated data sources and the ease of obtaining and querying them have already and will continue to unravel genetic and molecular underpinnings of autoimmune disease (37). An example are the currently ongoing 61 UKB-approved projects that are related to autoimmune disease (keyword search “autoimmune disease”, June 13th, 2022). Our survey shows that the catalog of autoimmune GWAS studies and associations is already very comprehensive and generated in more than a decade. PGS for autoimmune diseases are rather few, very novel and largely developed within the last three years. Accordingly, in the polygenic disease genetics field, research efforts go into two interrelated directions: (i) unraveling specific functional effects of variants and (ii) combining effect estimates for a better personalized risk prediction. Computational approaches toward the first aim are the association of risk alleles with molecular traits (38) and the identification of functional variants *via* so-called fine-mapping (39). Although there is progress in the field, it is still a long way from variant associations to molecular disease mechanisms as well as treatments (37). Toward the second aim, polygenic scores are still being improved, for example by considering rare variants (40) or inclusion of functional information (41). Optimizing prediction performance is non-trivial, since machine learning models need to be calibrated to generalize to unseen data, i.e. overfitting of training data prevented (42). The AUROC of current autoimmune disease PGS varies widely (range 0.56-0.99; typically 0.6-0.8). Further evaluation of polygenic scores in more cohorts and systematic comparisons, facilitated by the PGS catalog, will help gaining further insights into PGS predictive performance for individual autoimmune diseases. Perspectively, PGS can be amended with other biomedical, clinical and behavioral data. Such rich, combined data sources together with recent developments in artificial intelligence promise to improve prediction of disease and personalized treatment options (43). Finally, polygenic scores can be used to investigate interactions of genetic and environmental factors, which is particularly relevant for autoimmune diseases, in which environmental factors play a key role (44).

## Author contributions

RS and IW summarized data and generated tables and figures. RS, CF, and IW wrote the first draft of the manuscript. All authors contributed to writing and editing the manuscript. All authors approved the submitted version.

## Funding

HB and IW acknowledge funding by the Deutsche Forschungsgemeinschaft (DFG, German Research Foundation) under Germany’s Excellence Strategy—EXC 22167-390884018. This work was funded by the Research Training Group 2633 Autoimmune Pre-Disease project A9.

## Acknowledgments

The authors acknowledge support through the high-performance computer cluster (OMICS-Cluster) of the University of Lübeck.

## Conflict of interest

The authors declare that the research was conducted in the absence of any commercial or financial relationships that could be construed as a potential conflict of interest.

## Publisher’s note

All claims expressed in this article are solely those of the authors and do not necessarily represent those of their affiliated organizations, or those of the publisher, the editors and the reviewers. Any product that may be evaluated in this article, or claim that may be made by its manufacturer, is not guaranteed or endorsed by the publisher.
